# A statistical analysis protocol for the time-differentiated target temperature management after out-of-hospital cardiac arrest (TTH48) clinical trial

**DOI:** 10.1186/s13049-016-0334-0

**Published:** 2016-11-28

**Authors:** Hans Kirkegaard, Asger Roer Pedersen, Ville Pettilä, Jakob Hjort, Bodil Steen Rasmussen, Inge de Haas, Jørgen Feldbæk Nielsen, Susanne Ilkjær, Anne Kaltoft, Anni Nørgaard Jeppesen, Anders Morten Grejs, Christophe Henri Valdemar Duez, Alf Inge Larsen, Valdo Toome, Urmet Arus, Fabio Silvio Taccone, Christian Storm, Timo Laitio, Markus B Skrifvars, Eldar Søreide

**Affiliations:** 1 Department of Anaesthesiology and Intensive Care Medicine, Aarhus University Hospital, Aarhus, Denmark; 2 Research Center for Emergency Medicine, Aarhus University, Aarhus, Denmark; 3 Hammel Neurorehabilitation Centre and University Research Clinic, Aarhus University, Aarhus, Denmark; 4 Division of Intensive Care, Department of Anaesthesiology and Intensive Care Medicine, Helsinki University Hospital, Helsinki, Finland; 5 Division of Intensive Care, Department of Anaesthesiology and Intensive Care Medicine, Helsinki University, Helsinki, Finland; 6 Inselspital, Bern University Hospital, University of Bern, Bern, Switzerland; 7 Department of Clinical Medicine, Aarhus University, Aarhus, Denmark; 8 Department of Anaesthesiology and Intensive Care Medicine, Aalborg University Hospital, Aalborg, Denmark; 9 Department of Clinical Institute, Aalborg University, Aalborg, Denmark; 10 Hammel Neurorehabilitation Centre and University Research Clinic, Aarhus University, Aarhus, Denmark; 11 Department of Cardiology, Aarhus University Hospital, Aarhus, Denmark; 12 Department of Cardiology, Stavanger University Hospital, Stavanger, Norway; 13 Department of Clinical Science, University of Bergen, Bergen, Norway; 14 Department of Anaesthesiology, Intensive Care and Emergency Medicine, North Estonia Medical Centre, Tallinn, Estonia; 15 Department of Intensive Cardiac Care, North Estonia Medical Centre, Tallinn, Estonia; 16 Department of Intensive Care, Erasmus Hospital, Université Libre de Bruxelles (ULB), Brussels, Belgium; 17 Department of Internal Medicine, Nephrology and Intensive Care, Charité-Universitätsmedizin Berlin, Berlin, Germany; 18 Department of Anaesthesiology and Intensive Care, University of Turku, Turku, Finland; 19 Division of Perioperative Services, Intensive Care Medicine and Pain Management, Turku University Hospital, Turku, Finland; 20 Australian and New Zealand Intensive Care Research Centre, School of Public Health and Preventive Medicine, Monash University Melbourne, Monash, Australia; 21 Department of Anaesthesiology and Intensive Care, Stavanger University Hospital, Stavanger, Norway; 22 Department of Clinical Medicine, University of Bergen, Bergen, Norway

**Keywords:** Cardiac arrest, Heart arrest, Out-of-hospital, Targeted temperature management, Cerebral performance category, Mortality, Critical care, Intensive care, Randomised controlled trial

## Abstract

**Background:**

The TTH48 trial aims to determine whether prolonged duration (48 hours) of targeted temperature management (TTM) at 33 (±1) °C results in better neurological outcomes compared to standard duration (24 hours) after six months in comatose out-of-hospital cardiac arrest (OHCA) patients.

**Methods:**

TTH48 is an investigator-initiated, multicentre, assessor-blinded, randomised, controlled superiority trial of 24 and 48 hours of TTM at 33 (±1) ° C performed in 355 comatose OHCA patients aged 18 to 80 years who were admitted to ten intensive care units (ICUs) in six Northern European countries.

The primary outcome of the study is the Cerebral Performance Category (CPC) score observed at six months after cardiac arrest. CPC scores of 1 and 2 are defined as good neurological outcomes, and CPC scores of 3, 4 and 5 are defined as poor neurological outcomes. The secondary outcomes are as follows: mortality within six months after cardiac arrest, CPC at hospital discharge, Glasgow Coma Scale (GCS) score on day 4, length of stay in ICU and at hospital and the presence of any adverse events such as cerebral, circulatory, respiratory, gastrointestinal, renal, metabolic measures, infection or bleeding.

With the planned sample size, we have 80% power to detect a 15% improvement in good neurological outcomes at a two-sided statistical significance level of 5%.

**Discussion:**

We present a detailed statistical analysis protocol (SAP) that specifies how primary and secondary outcomes should be evaluated. We also predetermine covariates for adjusted analyses and pre-specify sub-groups for sensitivity analyses. This pre-planned SAP will reduce analysis bias and add validity to the findings of this trial on the effect of length of TTM on important clinical outcomes after cardiac arrest.

**Trial registration:**

ClinicalTrials.gov: NCT01689077, 17 September 2012

## Background

Time-differentiated targeted temperature management after out-of-hospital cardiac arrest (TTH48) is an international multicentre randomised pragmatic clinical trial. It is the first randomised trial to explore the influence of prolonged targeted temperature management (TTM) on neurological outcomes in out-of-hospital cardiac arrest (OHCA) patients. In 2002 two randomized studies demonstrated an effect on cerebral outcome of TTM at 33 °C for respectively 12 and 24 hours following OHCA. This led to international guidelines recommendations of the use of TTM. Animal studies have demonstrated that cooling for periods longer than 24 hours might even add to the beneficial effect. Furthermore in neonates it is good clinical practice to cool patients with anoxic brain injury for 72 hours. However we found no human interventional studies comparing different durations of TTM after cardiac arrest with return of spontaneous circulation (ROSC). Enrolment of patients began 16 February 2013, and the last of the 355 patients was included 1 June 2016. The complete six-month outcome data will be accessible in December 2016. The study protocol was published previously [1]. According to good clinical practice, and in order to prevent outcome reporting bias and data-driven analyses, it is recommended to prepare and publish a statistical analysis protocol (SAP) for the main trial before any data analyses are initiated [2, 3]. Thus, our detailed SAP was formulated while data were still being collected, and it was approved by the trial steering committee.

## Methods

### Trial overview

The TTH48 trial is an investigator-initiated, international, multicentre, outcome assessor-blinded, parallel group, pragmatic, randomised controlled trial (RCT) comparing TTM at 33 (+/-1) °C for 24 and 48 hours in OHCA patients.

The aim of TTH48 is to compare the effects of prolonged (48 hours) and standard duration (24 hours) of TTM at 33 (±1) °C (TTM33) in comatose OHCA patients. Our hypothesis is that 48 hours of TTM results in better neurological outcomes (primary outcome) and lower mortality (secondary outcome) without an increase in adverse effects. In line with previous TTM studies, the primary outcome is defined as the Cerebral Performance Category (CPC) score at six months after cardiac arrest, at this time the recovery potential following OHCA induced cerebral injury is fully exploited. The study comprises adult comatose patients with return of spontaneous circulation (ROSC) suffering from OHCA. The published trial protocol (version 6.3) [1] is available online at http://www.tth48.com In total, ten intensive care units (Aarhus, Helsinki, Aalborg, Tallinn, Stavanger, Brussels, Berlin, Copenhagen, Odense and Turku) in six Northern European countries participated in the study. The last trial patient was randomised on 1 June 2016. The protocol was prepared according to the current version of the Helsinki Declaration (2013) and approved by the local and regional research ethics committees listed in the appendix.

### Aim

To prepare a detailed statistical analyses protocol for the TTH48 trial.

We will also predetermine covariates, adjust analyses and select subgroups for analysis, and use the OHCA score, a validated score for predicting poor outcomes after OHCA [4, 5].

### Outcome

#### Primary outcome

The primary outcome is the CPC score at six months after cardiac arrest. As in the previous TTM trials, good neurological outcomes are defined as CPC scores of 1 or 2, and poor neurologic outcomes are defined as CPC scores of 3, 4 or 5 (death).

#### Secondary outcomes and adverse events

The secondary outcomes are as follows: 1) mortality within six months after cardiac arrest, 2) CPC score at hospital discharge, 3) Glasgow Coma Scale (GCS) score on day 4 after cardiac arrest, 4) adverse event(s), registered on day 4 and at discharge from hospital and defined as the occurrence of one or more events such as cerebral, circulatory, respiratory, gastrointestinal, renal, metabolic measures, infection or bleeding, 5) progression of GCS from days 1 to 7 in the ICU, 6) GCS score at ICU discharge and hospital discharge and 7) CPC score at 30 and 90 days after cardiac arrest. A complete list of adverse events is presented in the previously published protocol paper [1].

### Power analyses and randomisation

From a clinical point of view we considered an absolute difference of 15% between the treatment groups to be relevant. For sample size calculation an equation for comparing two rates was used. A centre effect was not taken into account. To detect a 15% absolute difference in good outcomes (CPC score of 1 or 2) between the two groups (two-sided) with 80% study power and a 5% significance level (alpha), 169 patients were randomly assigned each group (n = 338). To compensate for losses between follow-ups and withdrawals of consent, the sample size was increased by 5% (17 patients) forming a study population of 355 patients. The web-based clinical trials management system ”TrialPartner” was used for randomization and data collection. Patients were assigned (1:1) to the treatment regimens by block randomisation using randomly permuted block sizes of 6,4 and 2 - stratified in terms of trial centre, age (above and below 60 years) and initial rhythm (shockable or non-shockable). TrialPartner permits, with a personal log-in, 24 hour randomization. All actions in the system are logged. The investigators did not know the block sizes. Inclusion of patients was stopped on 1 June 2016, resulting in the inclusion of 355 patients.

### Variables

We pre-defined variables for adjusted analyses of the primary and secondary outcomes for all trial patients and the pre-defined subgroups. The predefined covariates are as follows: trial site, age, gender, initial cardiac rhythm, time to ROSC and bystander cardiopulmonary resuscitation (CPR). In addition, the analyses will be adjusted with any additional baseline variable revealing a significant difference between the treatment (TTM) groups. The pre-defined subgroups are based on the following criteria: age (below or above 60 years), first presenting cardiac rhythm (shockable or not shockable), time to ROSC (longer or shorter than 25 minutes [6]), bystander CPR (performed or not performed), methods of TTM (invasive or non-invasive), time to target temperature from ROSC (longer or shorter than 240 minutes [7]), OHCA score (more or less than 20 points) [4, 5] and study site (the site with the highest number of recruited patients compared to the other sites).

### Baseline variables

Gender
Age
Comorbidity
  - According to the APACHE II score definition [8]
    i. Liver cirrhosis
    ii. Chronic heart failure (New York Heart Association [NYHA] Classification 4)
    iii. Chronic obstructive, restrictive or vascular pulmonary disease (treated by medication)
    iv. Chronic dialysis
    v. Immuno-compromised state
  - Diabetes mellitus (treated with medication)
  - Previous acute myocardial infarction (AMI)
  - Previous percutaneous coronary intervention (PCI) or coronary artery bypass grafting (CABG)
  - Previous cardiac arrest
  - Previous stroke
  - Pre-morbid CPC
Pre-hospital variables
  - Location of cardiac arrest
    i. Home
    ii. Public place
    iii. Other
  - Witness of arrest by
    i. Civilian
    ii. Emergency medical service (EMS)
    iii. None or unknown
  - Bystander CPR
    i. No
    ii. Yes
      1. With automated external defibrillator (AED)
      2. Without AED
  - Shockable rhythm at EMS arrival
    i. Yes
    ii. No or unknown
  - Use of mechanical compression device
    i. Yes
    ii. No or unknown
  - Time from cardiac arrest to start of basic life support (no flow time)
  - Time from cardiac arrest to start of advanced life support
  - Time from cardiac arrest to return of spontaneous circulation (ROSC) (no flow time plus total time of basic and advanced life support [low flow time])
Time from ROSC to start of TTM
Time from ROSC to randomisation
Time from ROSC to target temperature (34 °C)
8.TTM method
  - Non-invasive
  - Invasive (intravenous TTM catheter)
  - Cold intravenous fluids
Data on ICU admission
  - First measured core temperature
  - First measured blood pressure (mean arterial pressure)
  - First measured pulse rate
  - First measured arterial PO2
  - First measured arterial PCO2
  - First measured arterial pH
  - First measured lactate
  - First measured serum creatinine
  - Acute ST-elevation infarction or new left bundle branch block (LBBB)

### Intervention period variables

Core temperature was measured in the bladder or oesophagus and reported each hour during the intervention period until 72 hours after the target temperature was achieved. The target temperature is defined as the time at which a core temperature of ≤ 34 °C is reached in the ICU. Time 0 (T0) is defined as the time when the target temperature is reached.

### Cardiovascular examinations and interventions

The number of patients during the first 72 hours of the study receiving coronary angiography (CAG) or percutaneous coronary intervention (PCI) will be reported. The number of patients receiving mechanical assist devices, including intra-aortic balloon pumps (IABPs); echocardiography; coronary bypass grafting; temporary pacemakers or implantable cardioverter devices (ICDs) will also be reported.

### Neurological examinations and interventions

The number of patients that undergo electro encephalograms (EEGs), somatosensory evoked potential tests (SSEPs), magnetic resonance imaging (MRI) and/or computed tomography of the cerebrum (CTC) during their initial ICU course will be reported. Further, the number of patients with diagnosed brain death and/or withdrawal of care due to a cerebral reason other than brain death will be reported.

### Other descriptive variables

The number of days in the ICU, days on mechanical ventilation (time to extubation) during index ICU admission and days in the primary hospital during index admission will be reported.

### Cause of death

Patients that die during the hospital stay will have their cause of death classified as one of the following: cardiac failure, multiple organ dysfunction, brain death or withdrawal of active treatment because of a cerebral condition other than brain death or another reason.

### General principles for the statistical analyses

The general principles of the analyses are as follows:

1. Analyses will be conducted according to the modified intention to treat (ITT) principle [9] if not otherwise stated.
2. Risks will be reported as hazard ratios (HR) or odds ratios (ORs) with a 95% confidence interval (CI).
3. All statistical tests will be two-sided with a significance level of 5%. Continuous variables will be summarised using mean +/- standard deviation (SD) for data with normal distribution and median with interquartile range points (25th and 75th) in parenthesis, for non-normally distributed data.
4. Missing values will not be imputed [10, 11]. The primary trial outcome will be tested in unadjusted analyses, and we do not foresee many missing values in these analyses.
5. Fisher’s exact test or the χ^2^ test will be used to compare binary or categorical data.
6. Continuous data will be tested for normality and compared between intervention groups using the Student t-test or Wilcoxon rank sum test according to the data distribution.
7. Unadjusted analyses and analyses adjusted for pre-defined covariates will be carried out for each primary and secondary outcome variables and for each sub-group [12]. Other analyses may be performed on different populations, such as per-protocol treated patients. The results of these additional analyses will be considered suitable for generating hypotheses only.
8. Logistic regression models will be used for multivariable analyses, and the results will be reported as ORs with 95% CI.
9. Cox regression analyses and a Kaplan Meier plot will be used for time-to-event analyses. Results will be given as hazard ratios (HRs) with 95% CI.
10. When blocks of many statistical tests are performed, the number of statistically significant results will be compared with the expected number of false significances due to statistical type I errors.

### Statistical analyses

#### Trial profile

**Fig. 1:**
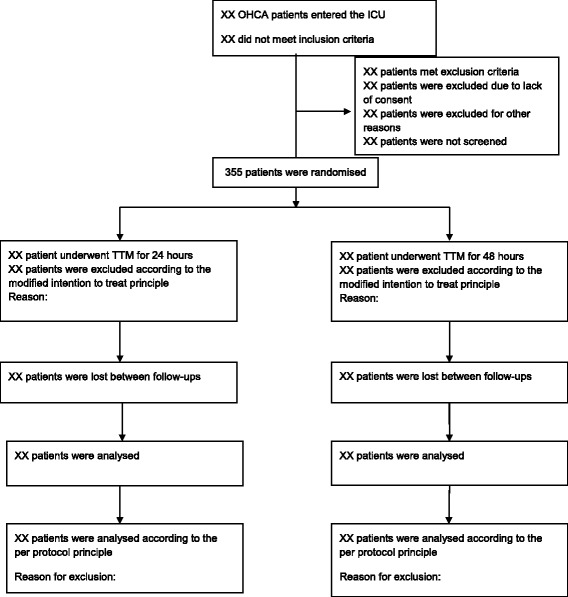
TTH48 CONSORT Flow Diagram

A CONSORT [13] diagram (Fig. 1) will display the flow of adult OHCA patients admitted to the ICU. The number of patients that did not meet inclusion criteria and the number of patients, excluded before and after randomisation, with reasons for exclusion will be reported.

### Primary outcome

The primary outcome is CPC score at six months after cardiac arrest. CPC scores of 1 and 2 indicate good neurological outcomes and scores of 3, 4 or 5 indicate poor neurological outcomes. The statistical null hypothesis is that there is no difference in primary outcomes between 24 and 48 hours of TTM. This will be tested in the modified ITT population by applying Fisher’s exact (or chi-square) test to a 2 × 2 contingency table.

The primary trial outcome will be reported as an unadjusted ratio with 95% CI and an absolute risk difference with 95% CI. Any significant difference in the number of patients needed to treat (NNT) for benefit or harm (with 95% CI) will be reported.

The second analysis of the primary outcome data, adjusted for the predetermined baseline covariates, will include all randomised patients who received the intervention and did not withdraw their consent according to the modified ITT principle [7, 10].

The third analysis of the primary outcome, adjusted for the predetermined baseline covariates, will include randomised patients who did not violate any major protocols (per-protocol analysis).

The fourth analysis of the primary outcome data will be adjusted for pre-specified covariates and any significant imbalance in baseline covariates (*P* < 0.05).

The fifth analysis will be a sensitivity analysis of pre-defined sub-groups (as described earlier).

All of these analyses will use logistic regression models. The goodness-of-fit of the logistic regression models will be assessed by graphical diagnostics and the Hosmer-Lemeshow test. The results will be reported as ORs with 95% CI.

### Secondary outcomes

#### Mortality

A time-to-event analysis will be performed for mortality (up to six months after cardiac arrest) using Kaplan-Meier curves, log-rank tests, unadjusted and adjusted Cox proportional hazard regression models and hazard ratios with 95% CI.

#### Adverse events

The number of patients that experience one or more adverse events and the number of patients that experience no adverse events will be compared using Fisher’s exact (or chi-square) test. If there is a significant difference between the treatment groups in terms of the occurrence of adverse events, we will try to identify the event that is responsible for the difference. Prolonged TTM may increase the risk of infection and, as it affects coagulation, the frequency of pneumonia and any bleeding in the two treatment groups will be compared using Fisher’s exact test. The definitions of pneumonia and bleeding are presented in the protocol paper [1].

#### GCS and CPC

GCS scores will be reported as the median and range and compared between the two treatment groups with a nonparametric test. CPC scores will be compared between the groups using logistic regression and following the modified ITT principle, as with the primary outcome data.

### Baseline variables

The baseline characteristics listed above will be presented in a table presenting the treatment groups. Discrete variables will be summarised according to frequencies and percentages. Percentages will be calculated according to the number of patients for which data are available. When values are missing, the actual denominators will be stated.

### Intervention period variables

#### One hour means of temperature in the two intervention groups will be displayed in a graph summarised according to distribution as mean +/- 2 SD

Temperatures from the target temperature (T0) until 24 hours of TTM will be compared using a repeated measures analysis of variance (ANOVA). The time from ROSC to achievement of the target temperature will be compared between the two groups using a parametric or nonparametric test according to the data distribution. Temperatures measured during the rewarming period will be compared between the two groups using a repeated measures ANOVA. The temperature change during the first four hours after rewarming to 37 °C will be compared between the two groups. The area under the temperature curve and the peak temperature will be calculated and compared using parametric or nonparametric statistics according to the data distribution. The time from the end of TTM (24 or 48 hours) to a temperature of 37 °C will be compared between the groups. The temperatures measured at 60 and 72 hours will also be compared between the two groups.

### Neurological, cardiovascular and other outcome data

Data will be presented in a table divided by treatment group. Discrete variables will be summarised according to frequencies and percentages. Percentages will be calculated according to the number of patients for which data are available. When values are missing, the actual denominators will be stated.

Patients in the 48 hour group will, according to the intervention, be sedated and placed on a ventilator 24 hours longer than the 24 hour group. This may, however, be balanced by the potential positive effect of prolonged TTM. In order to elucidate this interaction, the length of stay in the ICU, days on mechanical ventilation (time to extubation) and hospital length of stay (LOS) will be compared between groups. The data will be tested for normality and compared with Student’s t-test or a non-parametric test according to the data distribution.

### Data monitoring, interim analyses and safety

The trial has been monitored by the Data Monitoring and Safety Committee (DMSC), an independent committee that aims to safeguard the interests of trial participants, assess the safety and efficacy of interventions during the trial and monitor the overall conduct of the clinical trial. Two interim analyses were performed. The first was based on adverse events that occurred during the intervention performed on the first 175 patients, and the second was based on six-month CPC analyses of the same 175 patients. The DMSC used *P* < 0.001 (Haybittle-Peto) and group sequential monitoring boundaries as statistical limits to guide its recommendations regarding early termination of the trial. The DMSC recommended that the TTH48 trial should continue as planned following two data safety analyses (20 December 2015 and 27 February 2016). Tests for futility were not performed.

Data monitoring will be performed at each site by the principal investigator (PI) or a person appointed by the PI. The PI’s unit will be monitored by a member of the steering committee located outside of the PI’s centre. It will be ensured that all enrolled patients signed consent forms as per local ethical practice. A randomly chosen portion of trial patients (20%) will be checked for appropriate inclusion and exclusion criteria. The complete data of a randomly chosen sample (5%) of the included patients will be reviewed at each site.

When the final data have been registered, the trial database will be locked. Afterwards, the planned statistical analyses will be performed by a statistician using blinded binary indicators for the two treatment groups. The results will then be incorporated in the final report by the writing group before the data are un-blinded. Hans Kirkegaard (the study’s PI) and Asger Pedersen (the study’s statistician) will take responsibility for ensuring the integrity of the data. The study data will be made available to the journal to which the study is submitted.

### Figures and tables

Figure 1 will be a CONSORT flow chart. Figure 2 will be a temperature graph for the two groups with hours on the x-axis (-4 to 80, with 0 as the time at which the patients in each group reached a temperature of 34 °C in the ICU) and temperature on the y-axis. Figure 3 will be a Kaplan-Meier plot presenting the mortality rate during the six-month follow-up period. Figure 4 will be a forest plot of the effects of intervention on the pre-defined sub-groups. Table 1 will present baseline variables. Table 2 will present adverse events. Table 3 will present GSC and CPC scores, with the number of patients in each category of CPC reported separately but not compared. Table 4 will present the cardiovascular and neurological examinations and interventions, Table 5 will present patients’ ICU and in-hospital LOS and time to extubation and Table 6 will present the course of death of patients that died in the hospital.

## Conclusion

In order to avoid the risk of outcome reporting bias and data-driven analyses, the present paper reports a pre-defined detailed SAP for the TTH48 trial. The TTH48 trial aims to increase current knowledge regarding the optimal length of TTM and reduce the number of knowledge gaps referred to in the recommendations in the recent Consensus on Science and Treatment publication [14], one of which concerns the effect of the length of TTM.

## Abbreviations

AED: Automated external defibrillator
AMI: Acute myocardial infarction
ANOVA: Analysis of variance
CABG: Coronary artery bypass grafting
CAG: Coronary angiography
CI: Confidence interval
CPC: Cerebral Performance Category
CPR: Cardiopulmonary resuscitation
CTC: Computed tomography of the cerebrum
DSMC: Data and Safety Monitoring Committee
EEG: Electroencephalogram
EMS: Emergency medical service
GCS: Glasgow Coma Scale
HR: Hazard ratio
IABP: Intra-aortic balloon pump
ICD: Implantable cardioverter
ICU: Intensive care unit
ITT: Intension-to-treat
LBBB: Left bundle branch block
MRI: Magnetic resonance imaging
NNT: Number needed to treat
NYHA: New York Heart Association
OHCA: Out-of-hospital cardiac arrest
OR: Odds ratio
PCI: Percutaneous coronary intervention
PI: Principal investigator
RCT: Randomised controlled trial
ROSC: Return of spontaneous circulation
SAP: Statistical analysis protocol
SD: Standard deviation
SSEP: Somatosensory evoked potentials
TTM: Targeted temperature management
